# Endophthalmitis Caused by *Bacillus cereus*: Clinical Characteristics, Outcomes and Antibiotic Susceptibility

**DOI:** 10.3390/antibiotics13070658

**Published:** 2024-07-16

**Authors:** Jiayi Zheng, Liping Lin, Jingyu Liao, Xinlei Zhao, Jiaqi Lin, Fang Duan

**Affiliations:** State Key Laboratory of Ophthalmology, Zhongshan Ophthalmic Center, Sun Yat-sen University, Guangzhou 510060, China; zhengjiayi010@163.com (J.Z.); pear-0663@163.com (L.L.); fish857@163.com (J.L.); conberceptvegf@gmail.com (X.Z.); linjq55@mail3.sysu.edu.cn (J.L.)

**Keywords:** endophthalmitis, *Bacillus cereus*, visual outcomes, enucleation or evisceration outcomes, antibiotic susceptibility

## Abstract

*Bacillus cereus* endophthalmitis is a severe vision-threatening disease. This study aimed to analyze the clinical characteristics, antibiotic susceptibility, and risk factors for poor final visual acuity (VA) and enucleation or evisceration (ENEV) outcomes of *B. cereus* endophthalmitis patients. We retrospectively reviewed 52 cases (52 eyes) of culture-proven *B. cereus* endophthalmitis at Zhongshan Ophthalmic Center from January 2013 to December 2023. The mean age of the patients was 38.1 ± 20.1 years, and males composed the majority (90.4%) of the sample size; laborers (32.7%) and farmers (19.2%) were the primary occupations of the patients. All cases were caused by ocular trauma. Forty-one of 51 eyes (80.4%) had a final VA worse than the ability to count fingers (CFs), and 15 of the 52 total eyes (28.8%) underwent ENEV. Binary logistic forward (LR) regression analysis demonstrated that red eye (odds ratio [OR], 13.13; 95% confidence interval [CI], 1.58–108.80; *p* = 0.017), eye pain (OR, 22.87; 95% CI, 1.00–522.72; *p* = 0.050), and corneal edema/ulcer (OR, 13.13; 95% CI, 1.58–108.80; *p* = 0.017) were significant risk factors for poor VA outcomes. Conjunctival sac purulent discharge (OR, 10.08; 95% CI, 2.11–48.12, *p* = 0.004) and white blood cell (WBC) count (OR, 1.35; 95% CI, 1.06–1.72, *p* = 0.016) were significant risk factors for ENEV outcomes. *B. cereus* showed susceptibility rates of 100.0% to vancomycin and ofloxacin; 98.0% to levofloxacin; 93.3% to ciprofloxacin; 87.5% to imipenem; and 78.9% to tobramycin. The susceptibility to azithromycin and clindamycin was 66.7% and 50.0%, respectively. In contrast, *B. cereus* was resistant to penicillin (susceptibility at 3.8%), cefuroxime (5.6%), and cefoxitin (37.1%).

## 1. Introduction

Infectious endophthalmitis is characterized by intraocular inflammation caused by pathogens such as bacteria and fungi, posing an unfavorable threat to vision. Endophthalmitis is classified as exogenous or endogenous based on the source of the pathogen. Exogenous endophthalmitis primarily occurs due to the invasion of pathogens into the eye following intraocular surgery, penetrating trauma, or contiguous spread from adjacent tissues [1]. Ocular trauma-related endophthalmitis accounts for 49.6–58.0% of infectious endophthalmitis cases [2,3,4]. Gram-positive bacteria are the predominant pathogens responsible for endophthalmitis after ocular trauma (49.7–84.5%) [2,4,5].

*Bacillus cereus* is a group of gram-positive *Bacillus* that can persist and survive in harsh environments by forming endospores and biofilms. The resistance of bacterial spores to heat, cold, dryness, gamma rays, and ultraviolet radiation conferred strong survival capabilities and wide distribution. Additionally, *B. cereus* is highly virulent and produces a range of virulence factors, including pore-forming toxins, cereulide, hemolysins, enterotoxins, proteases, and phospholipases [6]. *B. cereus* endophthalmitis commonly occurs after ocular trauma, accounting for 5.1–18.5% of post-traumatic endophthalmitis cases [4,7,8]. However, due to its potent virulence, once in the eye *B. cereus* rapidly induces symptoms of endophthalmitis, typically within one day [9]. *B. cereus* endophthalmitis commonly causes poor visual outcomes and may require evisceration or enucleation (ENEV) [9,10,11]. Both are surgical procedures used to remove the eye, typically employed to treat severe ocular trauma and blind, painful eyes. Enucleation removes the entire globe, with an implant secured within the muscle cone. Evisceration, on the other hand, involves the removal of intraocular contents through a corneal or scleral incision, preserving the sclera, with an implant secured into the sclera [12,13]. Therefore, cases of *B. cereus* endophthalmitis urgently require early recognition and prompt treatment.

Previous research on *B. cereus* endophthalmitis had a relatively small sample size. Jean B. Vahey et al. reported 12 *B. cereus* endophthalmitis cases from 1974–1990 [10], and Don B. David et al. reported 38 cases of *B. cereus* endophthalmitis from 1981–1991 [14]. Therefore, in the present study, we retrospectively reviewed the clinical characteristics (including etiology, symptoms, signs, imaging and laboratory examinations, and treatment), intraocular isolates, and antimicrobial susceptibility results of 52 patients with culture-proven *B. cereus* endophthalmitis at our center from January 2013 to December 2023 and analyzed the risk factors for poor visual outcomes and ENEV outcomes. Based on our review of the relevant literature, this study represents the largest series of *B. cereus* endophthalmitis cases reported. These findings provide a reference for guiding the treatment of *B. cereus* infection.

## 2. Results

### 2.1. Patient Characteristics

A total of 52 eyes of 52 patients were included in the current study. The mean age of the patients was 38.1 ± 20.1 years (range, 3–74 years). The majority of patients were male (47/52, 90.4%), while females constituted 9.6% (5/52). The highest proportions of patients were laborers (17/52, 32.7%) and farmers (10/52, 19.2%). Preschool children and students constituted 19.2% (10/52) of the sample. All cases in our study were unilateral; the right eye was involved in 28 cases (53.8%), and the left eye was involved in 24 cases (46.2%). All patients had culture-positive *B. cereus* endophthalmitis identified by the VITEK 2 system, and 30.8% of patients (16/52) developed orbital cellulitis. The final visual acuity (VA) was determined for 51 patients (one patient was not cooperative for visual acuity examination) and was categorized into the favorable VA group, with a final VA equal to or better than the ability to count fingers (CFs), and the poor VA group, with a final VA worse than the ability to CFs. Ultimately, 28.8% of eyes (15/52) required ENEV. Depending on whether they underwent ENEV, we divided the patients into the ENEV group and the eye-sparing group.

In the favorable VA group, most of the patients were aged 0–20 years. Compared to the 0–20-years group, the proportion of patients with poor VA was greater in the >20-years age group, indicating that younger age correlates with better VA outcomes. Compared to preschool children and students, there was an increase in the proportion of laborers and farmers with poor VA outcomes. However, there was no significant difference in age or the proportion of occupations between the ENEV and eye-sparing groups. Sex did not significantly affect VA prognosis or the outcome of ENEV. The detailed information is shown in Table 1.

### 2.2. Etiology

Based on etiology, all cases in our study were attributed to ocular trauma, primarily caused by metal objects (including metal wires, metal nails or fragments, and scissors), accounting for the highest proportion of cases (48.1%, n = 25). Plant-related injuries accounted for 15.4% of the cases (n = 8), with 11.5% of cases caused by sharp plant sticks (n = 6), and 3.8% caused by wooden boards (n = 2). Injuries from lawn equipment accounted for 9.6% of the cases (n = 5), while 13.5% of the cases were due to impact by stones (n = 7), 3.8% by plastic fragments (n = 2), 1.9% by ceramic sheets (n = 1), and 1.9% by falls (n = 1). However, 5.8% of the cases (n = 3) were attributed to unidentified objects.

### 2.3. Clinical Features

As shown in Table 2, the mean length of hospital stay was 7.1 ± 3.2 days. Most patients received treatment within 24 h post-injuries (59.6%, 31/52). The mean follow-up time was 7.4 ± 17.1 months. The poor VA group had a longer hospitalization duration than the favorable VA group (*p* = 0.001). The ENEV group had a longer hospitalization duration than the eye-sparing group (*p* = 0.001). Treatment received within 24 h had no significant effect on VA or ENEV outcomes.

In this study, all cases were open globe injuries, 98.1% of cases (n = 51) were lacerations. Among them, 42.3% of cases (n = 22) were penetrations, and 55.8% were intraocular foreign bodies (IOFBs) (n = 29). One case was rupture (1.9%). The most common zone of injury was Zone I (90.4%), followed by Zone II (9.6%). The presence of IOFBs and the zone of injury had no significant effect on VA or ENEV outcomes.

Red eye (43/52, 82.7%) and eye pain (49/52, 94.2%) were the most common subjective symptoms in this context. Except for nine patients who were initially unable to cooperate during the visual examination, the initial VA of all patients was the ability to see hand movements (HMs) or below. The VA of HMs were observed in 12 cases (23.1%), 25 patients (48.1%) exhibited light perception (LP), and six patients (11.5%) exhibited no light perception (NLP). Intraocular hypertension, defined as intraocular pressure (IOP) > 21 mmHg or higher than normal measured by palpation, was documented in 12 patients (12/52, 23.1%) during the initial admission. The proportion of red eye was significantly greater in the poor VA group than in the favorable VA group (*p* = 0.001), while there was no significant difference between the ENEV group and the eye-sparing group (*p* = 0.090). The proportion of patients with intraocular hypertension in the ENEV group was significantly higher than that in the eye-sparing group (*p* = 0.017). All 12 patients with intraocular hypertension fell in the poor visual outcome group (100.0%); the intraocular pressure was higher in patients with poor visual outcomes but did not reach statistical significance (*p* = 0.150).

The common ocular signs identified in this study included conjunctival hyperemia and edema, purulent discharge of the conjunctival sac, corneal edema/ulcer, and hypopyon. Conjunctival edema was present in 26 eyes (26/52, 50.0%), and purulent discharge of the conjunctival sac was observed in 20 eyes (20/52, 38.5%). Corneal edema/ulcer was observed in 43 eyes (43/52, 82.7%), and hypopyon in 29 eyes (29/52, 55.8%). The ENEV group was more likely to have conjunctival edema and conjunctival sac purulent discharge than the eye-sparing group (*p* = 0.032 and *p* = 0.001, respectively). However, there was no significant difference between the good and the poor VA groups. The poor VA group more frequently presented with corneal edema/ulcer than the favorable group (*p* = 0.001), and the proportion in the ENEV group (100.0%) was greater than that in the eye-sparing group (75.7%), but the difference was not statistically significant (*p* = 0.090). No significant difference in hypopyon was detected in this study. Sixteen patients deteriorated to orbital cellulitis on admission (30.8%), and all of them were in the poor VA group (*p* = 0.045). The ENEV group had a greater proportion of orbital cellulitis than the eye-sparing group (*p* = 0.010).

Choroidal detachment and retinal detachment were detected by ocular B-scan ultrasonography and were observed in 42.3% (22/52) of the patients. Patients who underwent ENEV had a higher white blood cell (WBC) count (peripheral blood) than those who received eye-sparing therapy (*p* = 0.009), with no significant influence on the final VA. Poor VA outcomes and ENEV outcomes were likely to be associated with B-scan-detected choroidal detachment (*p* = 0.028 and *p* = 0.003, respectively). The proportion of retinal detachment detected by B-scan did not differ among patients in this study.

The Ocular Trauma Score (OTS) is used to standardize the assessment of ocular trauma and has prognostic value for open globe injuries [15,16]. This study’s most common OTS variables included endophthalmitis (100.0%) and retinal detachment (42.3%). The OTS values were calculated for 36 eyes during the initial examination (excluding nine cases that were not cooperative for the initial visual examination, and seven cases with invisible retinal situations and unavailable B-scan results). The OTS values were categorized, with 20 cases (38.5%) classified as OTS Category 1 and 16 cases (30.8%) as OTS Category 2. The OTS did not significantly differ between the poor VA group and the favorable VA group or between the ENEV group and the eye-sparing group, as presented in Table 2.

All patients, once clinically diagnosed with endophthalmitis, received intravenous antibiotic treatment. Overall, 67.3% of the patients received intravenous levofloxacin, 78.8% of the patients received cephalosporins, and 23.1% of the patients received intravenous vancomycin. Additionally, 86.5% of the patients underwent intravenous corticosteroid therapy. Subconjunctival injections of tobramycin and dexamethasone were administered to 14 eyes (26.9%), and tobramycin alone was administered to one eye (1.9%). All patients received topical anti-inflammatory and antibiotic therapy. According to the surgical treatment provided after the injury, all patients received open globe repair. Only 3.8% of cases (2/52) underwent open globe repair alone due to orbital cellulitis. Pars plana vitrectomy (PPV) was employed in 59.6% of cases (31/52). Among them, silicone oil was injected in 24 patients (46.2%), and intraocular antibiotics were injected into 11.5% (6/52) of these cases. Nine patients (17.3%) received intraocular antibiotics for corneal opacity. Fifteen patients (28.8%) required enucleation/evisceration. Among these, nine cases were for uncontrolled infection, and six with phthisis bulbi underwent the procedure to improve appearance, which occurred on average 2.8 months post-injury.

The type of intravenous antibiotic, intravenous corticosteroid therapy, subconjunctival antibiotic injection, and intraocular antibiotic injection did not significantly differ between the poor VA group and the favorable VA group or between the ENEV group and the eye-sparing group. All patients in the favorable final VA group underwent PPV, which was significantly higher than the percentage in the poor VA group (100.0% vs. 48.8%, *p* = 0.010). Similarly, PPV treatment was performed more frequently in the eye-sparing therapy group than in the ENEV group (81.1% vs. 6.7%, *p* < 0.001).

### 2.4. Risk Factors Associated with Poor Visual Acuity Outcomes and Enucleation or Evisceration

Variables with *p* < 0.1 in the univariate analysis were included in the binary logistic forward (LR) regression analysis (prolonged hospitalization was excluded as it was not an actual prognostic factor, and only indicated the severity of the condition). The multivariate analysis results presented in Table 3 revealed that red eye (odds ratio [OR], 13.13; 95% confidence interval [CI], 1.58–108.80; *p* = 0.017), eye pain (OR, 22.87; 95% CI, 1.00–522.72; *p* = 0.050), and corneal edema/ulcer (OR, 13.13; 95% CI, 1.58–108.80; *p* = 0.017) were significant risk factors for poor VA outcomes. Conjunctival sac purulent discharge (OR, 10.08; 95% CI, 2.11–48.12, *p* = 0.004) and WBC count (OR, 1.35; 95% CI, 1.06–1.72, *p* = 0.016) were significant risk factors for ENEV outcomes.

### 2.5. Susceptibility

Among the 52 *B. cereus* isolates in this study, 38 were from the vitreous culture, eight were from the aqueous culture, three were from the cornea, and three were from the conjunctival sac secretions. Notably, in 10 patients, other bacteria or fungi were concurrently cultured (bacteria included Lactococcus lactis, Enterococcus durans and Serratia marcescens; fungi included Aspergillus niger and mucormycosis). The susceptibility results of *B. cereus* are listed in Table 4. Only 3.8% of the *B. cereus* strain was sensitive to penicillin. Among cephalosporins, 5.6% of *B. cereus* isolates were sensitive to cefuroxime, and 37.1% were sensitive to cefoxitin. For aminoglycosides, the susceptibility was 53.8% to neomycin, 78.9% to tobramycin, and 75.7% to amikacin. Among quinolones, 100.0% were sensitive to ofloxacin, 98.0% were sensitive to levofloxacin, and 93.3% were sensitive to ciprofloxacin. In addition, the susceptibility rates were 100.0% for vancomycin, 87.5% for imipenem, 66.7% for azithromycin, and 50.0% for clindamycin. The susceptibility data are summarized in Table 4.

## 3. Discussion

In the present study, we reviewed 52 patients with culture-proven *B. cereus* endophthalmitis and found that the VA outcomes were poor and were associated with red eye, eye pain, and corneal edema/ulcer. Sixteen patients (30.8%) developed orbital cellulitis. Fifteen patients (28.8%) required ENEV, which was associated with conjunctival sac purulent discharge and high WBC count. These prognostic factors can assist clinicians in the early identification of patients who will develop a poor final VA prognosis and those potentially requiring ENEV surgery.

In our study, all patients experienced trauma, which is consistent with previous reports that *B. cereus* most commonly occurs following ocular trauma [4,10,11,14,17]. Males accounted for the highest proportion of patients with *B. cereus* endophthalmitis (90.4%), consistent with previous reports [11]. Laborer (32.7%) and farmer (19.2%) were the primary occupations, which is consistent with previous reports on traumatic endophthalmitis [18]. Among patients aged 18–50, the most common cause of injury was a metal wire or fragment (57.7%), corresponding to 53.8% of laborers in this age group. It should be noted that 20.0% of patients under ten years old were injured by bamboo sticks used for eating, which highlights the possibility of children being injured while using sharp tools for eating, underscoring the importance of preventing such occurrences.

Patients with bacterial endophthalmitis may experience partial or complete vision loss within hours to days. Due to the potent virulence of *B. cereus*, endophthalmitis caused by this bacterium progresses even more rapidly [19]. In our study, a large proportion of the patients (59.6%) presented within 24 h, consistent with previous reports [9]. Despite receiving ocular treatment at an earlier stage, the visual prognosis for *B. cereus* endophthalmitis was still extremely poor, with 10 patients having a final visual acuity ≥ the ability to CFs, 10 patients able to see HMs, seven patients having LP, and 25 patients having NLP, similar to previous reports [9,10,20,21,22]. In our study, the incidence rate of ENEV was 28.8% (15/52), lower than previously reported (57.9–75.0%) [10,12], which may be attributed to the advancements and widespread adoption of PPV in recent decades.

We found poor VA outcomes were associated with red eye, eye pain, and corneal edema/ulcers. Red eye and eye pain were typical symptoms of endophthalmitis [23]. Red eye and eye pain were identified as poor visual prognostic factors in previous reports on *Klebsiella pneumoniae* endophthalmitis, consistent with our results [24]. In this study, the causes of red eye and eye pain included mechanical injury or inflammatory reactions following ocular trauma and endophthalmitis associated with bacterial virulence. Persistent red eye and eye pain indicated the ongoing activity of endophthalmitis and sustained activity of *B. cereus*. *B. cereus* exhibits powerful virulence and induces severe inflammatory responses. It can kill corneal stromal cells in vitro within 6 h [25]. The disruption of corneal transparency hampers the visual field of observing posterior structures of the eyeball, impeding the use of PPV as a treatment method.

In cases where the patient’s condition deteriorates with uncontrollable infection, surgery for ENEV as treatment is needed. The presence of conjunctival sac purulent discharge is one of the risk factors for ENEV outcomes, a relationship not previously reported. Additionally, our study revealed many cases of conjunctival purulent discharge accompanied by corneal ulceration and dissolution (13/20, 65.0%). This may be due to a higher bacterial load and increased virulence of the bacterial strains in cases where conjunctival sac purulent discharge was present, leading to more challenging infection control and necessitating ENEV. Furthermore, the WBC count upon admission was identified as a risk factor for ENEV prognosis. The WBC count is a commonly used biomarker for inflammation. In this study, a higher white blood cell count reflects more severe inflammation and a more challenging control of inflammatory conditions, thereby increasing the likelihood of requiring ENEV.

PPV surgery is considered an important method for treating endophthalmitis and improving visual outcomes in recent decades [26,27,28,29]. According to our univariate analysis, the PPV was significantly greater in the favorable VA group and eye-sparing group than in the poor VA group and ENEV group. Therefore, we speculate that PPV is associated with favorable VA outcomes. It is noted that intravitreal antibiotic injection is also an alternative choice for treating endophthalmitis; however, the PPV can effectively reduce the need for additional treatment beyond intravitreal antibiotic injection [30]. In our study, nine patients underwent only intraocular injections of antibiotics due to severe corneal opacification, leading to missed opportunities for PPV. Therefore, the current challenge is the narrow window for PPV in *B. cereus* endophthalmitis.

Sixteen patients developed orbital cellulitis (30.8%), and 81.3% of them were male, consistent with the previously reported proportion of males [31,32]. In previous reports on orbital cellulitis, *B. cereus* was identified as the predominant pathogen (18.8%) [32], indicating a greater propensity for *B. cereus* endophthalmitis to advance to orbital cellulitis. The incidence of orbital cellulitis in the univariate analysis was significantly higher in the poor VA and poor ENEV groups than in the favorable VA and eye-sparing groups (*p* = 0.045 and *p* = 0.010, respectively). Our study revealed that 56.3% of patients (9/16) required ENEV, higher than the 40.5% reported in a previous study on orbital cellulitis with diverse causative pathogens [32]. This difference may be due to the stronger virulence of *B. cereus*.

The OTS has shown an accuracy of 80% in predicting visual outcomes six months post-injury [33]. In our study, only OTS 1 and 2 were observed, demonstrating that we encountered severe cases of aggressive and rapidly progressive *B. cereus* endophthalmitis. The low OTS in this study was attributed to two main reasons: first, all cases were endophthalmitis, and second, a significant portion of cases exhibited retinal detachment. Considering that in this study, the majority of injuries were located in Zone I, the virulence of *B. cereus* may play a crucial role in retinal detachment. Previous studies have shown that *B. cereus* can cause a reduction in the retinal response detectable by ERG as early as 3 h post-infection [34] and can kill retinal Müller cells in vitro within 6 h [25]. This suggests that poor visual acuity in our study is not only attributed to the nature of the injury but also to the potent virulence of *B. cereus*.

Previous studies on *B. cereus* susceptibility have shown high susceptibility to imipenem, vancomycin, and ciprofloxacin. However, *B. cereus* is resistant to penicillin and some cephalosporins [35]. Similarly, in our study, *B. cereus* showed susceptibility rates of 100.0% to vancomycin and ofloxacin, 98.0% to levofloxacin, 87.5% to imipenem, and 78.9% to tobramycin. In contrast, these strains were resistant to penicillin (susceptibility at 3.8%), cefuroxime (5.6%), and cefoxitin (37.1%).

This study has certain limitations. First, due to the rarity of *B. cereus* endophthalmitis, the sample size was relatively small, and conducting prospective studies was challenging. Second, this was a retrospective study with limitations due to its nature. Additionally, incomplete medical records and missing data led to the exclusion of some variables from the multivariable regression analysis. Nevertheless, our study provides valuable data on the risk factors for adverse outcomes and antibiotic susceptibility.

## 4. Materials and Methods

Retrospective and consecutive analyses were conducted on the medical records of all patients who were confirmed to have *B. cereus* endophthalmitis and admitted to hospitals at Zhongshan Ophthalmic Center, Guangzhou, China, from January 2013 to December 2023. This retrospective study design waived the requirement for patient consent. The study adhered to the principles of the Helsinki Declaration and was approved by the Institutional Ethics Committee of the Zhongshan Ophthalmic Center, Sun Yat-sen University.

### 4.1. Classification

The ocular trauma classification and definitions were based on the Birmingham Eye Trauma Terminology (BETT). All cases in this study involved open globe injuries (OGI). The classification of injury zones is as follows: for cases with multiple wounds, the final wound is considered definitive; for IOFBs, the entry point of the wound is definitive; for perforations, the exit point of the wound is definitive. Specifically, Zone I injuries are confined to the corneal and limbal area; Zone II injuries occur within 5 mm posterior to the limbus; Zone III injuries are located posteriorly to Zone II. The Ocular Trauma Score (OTS) was calculated using the previously reported method [16]. The OTS raw points were calculated based on the following variables: initial vision, rupture, endophthalmitis, perforating injury, retinal detachment, and afferent pupillary defect. Raw points were then converted into an OTS category as follows: OTS 1 scores range 0–44; OTS 2 scores range 45–65; OTS 3 scores range 66–80; OTS 4 scores range 81–91; OTS 5 scores range 92–100.

### 4.2. Procedures

We obtained the clinical characteristics of the patients through the electronic medical records system, including patient demographics, length of hospital stays, etiology, ocular conditions, examination results, management records, and visual outcomes at the last follow-up. The clinical diagnosis of endophthalmitis was primarily correlated with clinical manifestations, symptoms such as red eye, eye pain, and decreased vision, and ocular signs such as corneal edema/ulcer and anterior chamber and vitreous inflammation. *B. cereus* was confirmed through laboratory culture and identified using the VITEK 2 system. All patients received systemic and topical antibiotics immediately after clinical diagnosis, with antibiotic selection and surgical treatment strategies determined by the physician. Antibiotic selection was adjusted according to the results of pathogen identification. Surgical treatment included subconjunctival antibiotic injection, intraocular antibiotic injection, pars plana vitrectomy, and ENEV. The antibiotic used for subconjunctival injection was tobramycin, and the antibiotic used for intraocular injection was vancomycin.

### 4.3. Pathogen Identification and Antibiotic Susceptibility

Pathogen culture and identification: the specimens for this study were obtained from vitreous humor, aqueous humor, corneal scraping, and conjunctival discharge. Isolates were cultured on Columbia blood agar plates, followed by colony selection with similar morphologies. Sufficient pure culture colonies were suspended in sterile saline. The bacterial suspension was transferred into the bacteria-specific identification device, and the bacterial bio-type was analyzed by the VITEK 2 compact system (BioMérieux, Inc., Marcyl’ Étoile, France) with VITEK^®^ BCL identification cards following the manufacturer’s instructions.

Antimicrobial susceptibility testing: following the Clinical and Laboratory Standards Institute (CLSI) guidelines (https://www.clsi.org/, accessed on 2 October 2015), antimicrobial susceptibility testing was conducted using broth microdilution and the Kirby–Bauer disc diffusion method. (1) Using the broth microdilution method, the isolates were transferred to sterile inoculation water and adjusted to 0.5 McFarland standards, and the standardized bacterial fluid and the cation-adjusted Mueller–Hinton broth were mixed completely in the ratio of 1:200. An equivalent bacterial fluid mixture (100 µL) was added to wells of culture plates containing gradients of different antibiotic concentrations and antimicrobial susceptibility reagents. The plates were incubated at 35 °C for 16–20 h. Wells without precipitation and color change indicated no bacterial growth. The minimum inhibitory concentration (MIC), the lowest concentration at which bacterial growth was inhibited, was recorded and interpreted as “sensitive”, “intermediate”, or “resistant” according to the guidelines of the CLSI. The following antibiotics were tested in susceptibility analysis: penicillin (testing concentration at 0.064–8 μg/mL), cephalosporins (cefuroxime and cefoxitin, 0.5–64 μg/mL), fluoroquinolones (levofloxacin and ofloxacin, 0.032–4 μg/mL), aminoglycosides (neomycin, tobramycin, and amikacin, 0.25–32 μg/mL), imipenem (0.125–16 μg/mL), vancomycin (0.5–64 μg/mL), azithromycin (0.125–16 μg/mL), and clindamycin (0.125–16 μg/mL). (2) Using the Kirby–Bauer disk diffusion method, isolated colonies were transferred to saline solution and adjusted to 0.5 McFarland standards. Sterile cotton swabs were used to inoculate Mueller–Hinton (M–H) agar plates. The plates were rotated three times, approximately 60 degrees each time, to ensure even distribution of the inoculum. Antibiotic discs containing gradients of different concentrations were placed on the agar surface with at least a 24 mm gap between them. The plates were then incubated at 35 °C for 16–18 h (extended to 24 h for vancomycin). The diameter of the area without obvious bacterial growth was measured to determine the sensitivity of bacteria to antibiotics. We referenced the CLSI guidelines of breakpoints in various Gram-positive bacteria and our experience to interpret the antimicrobial susceptibility to provide advisory opinions for clinical treatment.

### 4.4. Definition

In this study, poor final visual acuity (VA) was defined as worse than that of the ability to count fingers (CFs), and favorable final visual acuity (VA) was defined as an ability to count fingers (CFs) or better. Enucleation or evisceration (ENEV) was defined as enucleation or evisceration surgery until the end of the follow-up period, while eye-sparing therapy was defined as not undergoing enucleation or evisceration surgery.

### 4.5. Statistical Analysis

The missing data rates were 28.8% (occupation), 13.5% (retinal detachment detected by B-scan), 13.5% (choroidal detachment detected by B-scan), 30.8% (OTS), and 9.6% (initial intraocular pressure). Samples with missing values were excluded from univariate analysis, and variables with missing values were excluded from multivariate analysis. Continuous variables were summarized using means and standard deviations, and categorical variables were summarized using percentages. Kruskal–Wallis tests were used to analyze continuous variables, chi-square tests were used to analyze categorical variables, and multivariable binary logistic regression analysis was conducted to assess prognostic factors for final visual outcomes and ENEV outcomes. Due to the association between corneal transparency and treatment selection, treatment-related variables were not included as independent variables in the multivariate analysis. All analyses were performed using SPSS (version 25.0; IBM, Armonk, NY, USA).

## 5. Conclusions

The proportion of patients with *B. cereus* endophthalmitis who underwent enucleation or evisceration decreased compared to previous reports. However, the visual outcome for these patients remains poor, with only 19.6% achieving visual acuity of CFs or better. The antibiotics that can be chosen for treating *B. cereus* endophthalmitis include vancomycin, ofloxacin, levofloxacin, and ciprofloxacin.

## Figures and Tables

**Table 1:** Comparison of demographics for visual acuity outcomes and enucleation/evisceration outcomes.

Factors		Ability to CFs or Better (n = 10)	Worse than the Ability to CFs (n = 41)	*p* Value	Enucleation/Evisceration (n = 15)	Eye-Sparing Therapy (n = 37)	*p* Value
Age (%)	0–20	6 (60.0)	4 (9.8)	0.006	1 (6.7)	10 (27.0)	0.304
	21–40	2 (20.0)	10 (24.4)		5 (33.3)	7 (18.9)	
	41–60	2 (20.0)	22 (53.7)		7 (46.7)	17 (45.9)	
	>60	0 (0.0)	5 (12.2)		2 (13.3)	3 (8.1)	
Sex (%)	Male	10 (100.0)	37 (90.2)	0.573	14 (93.3)	33 (89.2)	1.000
	Female	0 (0.0)	4 (9.8)		1 (6.7)	4 (10.8)	
Occupation (%)	Preschool and student	6 (60.0)	3 (7.3)	0.012	1 (6.7)	9 (24.3)	0.118
	Laborer	2 (20.0)	15 (36.6)		8 (53.3)	9 (24.3)	
	Farmer	2 (20.0)	8 (19.5)		2 (13.3)	8 (21.6)	

CFs = counting fingers.

**Table 2:** Comparison of clinical features for visual acuity outcomes and enucleation/evisceration outcomes.

Factors	The Ability to CFs or Better (n = 10)	Worse than the Ability to CFs (n = 41)	*p* Value	Enucleation/Evisceration (n = 15)	Eye-Sparing Therapy (n = 37)	*p* Value
Hospital stay (days)	4.6 ± 2.0	7.9 ± 3.1	0.001	9.3 ± 1.8	6.2 ± 3.3	0.001
Time from injury to treatment						
≤24 h	7 (70.0)	24 (58.5)	0.761	7 (46.7)	24 (64.9)	0.226
>24 h	3 (30.0)	17 (41.5)		8 (53.3)	13 (35.1)	
IOFB (%)	3 (30.0)	26 (63.4)	0.119	8 (53.3)	21 (56.8)	0.822
Injury zone						
Zone I	9 (90.0)	37 (90.2)	1.000	13 (86.7)	34 (91.9)	0.952
Zone II	1 (10.0)	4 (9.8)		2 (13.3)	3 (8.1)	
Red eye (%)	4 (40.0)	38 (92.7)	0.001	15 (100.0)	28 (75.7)	0.090
Eye pain (%)	8 (80.0)	40 (97.6)	0.094	15 (100.0)	34 (91.9)	0.548
Ocular hypertension (%)	0 (0.0)	12 (29.3)	0.150	7 (46.7)	5 (13.5)	0.017
Conjunctival edema (%)	3 (30.0)	22 (53.7)	0.323	11 (73.3)	15 (40.5)	0.032
Conjunctival sac purulent discharge (%)	2 (20.0)	18 (43.9)	0.304	11 (73.3)	9 (24.3)	0.001
Corneal edema/ulcer (%)	4 (40.0)	38 (92.7)	0.001	15 (100.0)	28 (75.7)	0.090
Hypopyon (%)	4 (40.0)	25 (61.0)	0.398	10 (66.7)	19 (51.4)	0.314
Lens damage (%)						
Orbital cellulitis	0 (0.0)	16 (39.0)	0.045	9 (60.0)	7 (18.9)	0.010
WBC count (×10^8^)	11.2 ± 3.7	12.7 ± 3.9	0.537	14.7 ± 4.2	11.4 ± 3.2	0.009
Retinal detachment (%)	1 (10.0)	20 (48.8)	0.230	10 (66.7)	12 (32.4)	0.092
Choroidal detachment (%)	0 (0.0)	22 (53.7)	0.028	12 (80.0)	10 (27.0)	0.003
OTS category						
Category 1	1 (10.0)	19 (46.3)	1.000	10 (66.7)	10 (27.0)	0.126
Category 2	1 (10.0)	15 (36.6)		4 (26.7)	12 (32.4)	
Subconjunctival antibiotics injection (%)	5 (50.0)	10 (24.4)	0.228	2 (13.3)	13 (35.1)	0.217
Intraocular antibiotics injection (%)	0 (0.0)	15 (36.6)	0.059	4 (26.7)	11 (29.7)	1.000
Pars plana vitrectomy (%)	10 (100.0)	20 (48.8)	0.010	1 (6.7)	30 (81.1)	<0.001

**Table 3:** Multivariate analysis of independent risk factors associated with poor visual acuity outcomes and enucleation or evisceration.

Factors	Odds Ratio	95% Confidence Interval	*p* Value
For poor visual acuity outcomes			
Red eye	13.13	[1.58–108.80]	0.017
Eye pain	22.87	[1.00–522.72]	0.050
Corneal edema/ulcer	13.13	[1.58–108.80]	0.017
For enucleation or evisceration			
Conjunctival sac purulent discharge	10.08	[2.11–48.12]	0.004
WBC count (×10^8^)	1.35	[1.06–1.72]	0.016

**Table 4:** Antibiotic susceptibility of *B. cereus* isolates.

Antibiotics	Sensitive n/N (%)	Intermediate n/N (%)	Resistant n/N (%)
Penicillin	2/52 (3.8%)	0/52 (0.0%)	50/52 (96.2%)
Cefuroxime	2/36 (5.6%)	1/36 (2.8%)	33/36 (91.7%)
Cefoxitin	13/35 (37.1%)	3/35 (8.6%)	19/35 (54.3%)
Neomycin	7/13 (53.8%)	6/13 (46.2%)	0/13 (0.0%)
Tobramycin	30/38 (78.9%)	5/38 (13.2%)	3/38 (7.9%)
Amikacin	28/37 (75.7%)	5/37 (13.5%)	4/37 (10.8%)
Ofloxacin	38/38 (100.0%)	0/38 (0.0%)	0/38 (0.0%)
Levofloxacin	50/51 (98.0%)	0/51 (0.0%)	1/51 (2.0%)
Ciprofloxacin	14/15 (93.3%)	1/15 (6.7%)	0/15 (0.0%)
Vancomycin	14/14 (100.0%)	0/14 (0.0%)	0/14 (0.0%)
Imipenem	14/16 (87.5%)	1/16 (6.3%)	1/16 (6.3%)
Azithromycin	10/15 (66.7%)	2/15 (13.3%)	3/15 (20.0%)
Clindamycin	6/12 (50.0%)	3/12 (25.0%)	3/12 (25.0%)

## Data Availability

The authors confirm that the data supporting the findings of this study are available within the article. Further inquiries can be directed to the corresponding authors.
